# From Bench to Bedside: Understanding General Anesthetics' Impact on PTSD

**DOI:** 10.2174/011570159X402072251103071408

**Published:** 2026-03-31

**Authors:** Ke Li, Meimei Guo, Wei Wei, Xiang Li, Mian Peng

**Affiliations:** 1 Department of Anesthesiology, Zhongnan Hospital of Wuhan University, Wuhan, China;; 2 Department of Neurosurgery, Zhongnan Hospital of Wuhan University, Wuhan, China;; 3 Brain Research Center, Zhongnan Hospital of Wuhan University, Wuhan, China;; 4 Frontier Science Center for Immunology and Metabolism, Wuhan University, Wuhan, China;; 5 Medical Research Institute, Wuhan University, Wuhan, China;; 6 Sino-Italian Ascula Brain Science Joint Laboratory, Zhongnan Hospital of Wuhan University, Wuhan, China

**Keywords:** PTSD, stress, fear memory, extinction memory, inhaled anesthetics, intravenous sedative

## Abstract

Post-traumatic stress disorder (PTSD) is a multifaceted mental health disorder arising from exposure to traumatic events, characterized by persistent and distressing symptoms. Recent years have seen growing recognition of anesthesia's dual role in PTSD. While countless patients receive anesthetics during traumatic medical care, their long-term psychological impacts remain unclear, raising urgent clinical questions. This review provides an in-depth analysis of both clinical and animal studies examining the effects of commonly used inhaled and intravenous anesthetics, such as sevoflurane/isoflurane, benzodiazepines (BZDs), ketamine, propofol, and dexmedetomidine (Dex), on PTSD. These studies reveal a complex interplay between these anesthetic agents and PTSD, highlighting their potential to either alleviate or exacerbate symptoms depending on various factors such as dosage, timing, and individual susceptibility. Additionally, this review synthesizes the current understanding of the underlying neurobiological mechanisms through which these anesthetics affect fear memory and neuroplasticity, offering valuable insights into their potential applications and limitations in managing PTSD.

## 1 INTRODUCTION

 Post-traumatic stress disorder (PTSD) is a debilitating mental illness that affects a minority of individuals following major traumatic events [1]. Its high comorbidity with major depressive disorder, elevated suicide risk, and significant psychosocial impairment contribute to a heavy social burden [2]. Despite its recognition as a critical public health challenge, current therapeutic interventions—primarily psychotherapy (*e.g*., CBT, exposure therapy) and pharmacotherapy—are only partially effective and exhibit limitations. While psychotherapies are the first line of treatment, their nonresponse rates approach 50% due to comorbidities and patient heterogeneity [3]. Pharmacological options show marginal efficacy, with delays in effect, side effects, and inconsistent guideline recommendations [4-6]. Consequently, more effective pharmacotherapies are urgently needed.

An acute traumatic period is often accompanied by the formation of fear memory, leading to the development of PTSD [7]. Dysfunction of the fear memory system underlies PTSD in perioperative and intensive care patients [8]. Trauma patients inevitably receive sedation, analgesia, or anesthesia in the emergency room, operating room, and intensive care unit. As we know, general anesthetics and sedatives influence the memory process by acting on neurotransmitters and receptors in specific circuits such as the adrenergic circuit [9]. Therefore, anesthetics and sedatives may impact fear memory and the development of PTSD. Previous studies have demonstrated evidence of the potential therapeutic effects of anesthetics on psychiatric disease [10-12]. However, studies investigating the effects of anesthetics on PTSD—particularly from a pathophysiological perspective—remain limited and require systematic synthesis. A critical gap exists in understanding the precise neurobiological mechanisms by which various anesthetics modulate fear memory processes, including formation, consolidation, and extinction.

 This review focuses on preclinical (rodent models) and clinical (human perioperative/ICU) studies linking anesthetics to PTSD-related fear memory, with an emphasis on studies from 2000 to 2024. The discussion encompasses mechanistic animal studies, RCTs, and observational clinical studies, prioritizing representative examples that offer translational insights. We summarize exhaustive clinical and preclinical evidence on the impacts of commonly used inhalational and intravenous anesthetics, including sevoflurane/ isoflurane, benzodiazepines (BZDs), ketamine, propofol, and dexmedetomidine (Dex), on the occurrence and development of PTSD. Unlike existing reviews that have primarily focused on clinical outcomes, our review uniquely integrates the pharmacological properties of these agents with their effects on discrete fear memory processes, which include acquisition, consolidation, extinction, reconsolidation, and forgetting processes at pathophysiological levels. Our aim is threefold: first, to encourage scholarly engagement with the prevention and treatment of PTSD through the lenses of anesthetic pharmacology and neurobiology; second, to provide clinicians with a robust framework for informed anesthetic selection; and third, to introduce novel perspectives and insights that may advance the field of anesthetic therapy.

## 2 METHOD

In this study, a narrative review methodology is employed, aiming to provide a comprehensive and critical synthesis of the existing literature on the effects of anesthetics on PTSD. A systematic literature search was performed to ensure transparency and minimize selection bias, although the full protocol for a systematic review (*e.g*., dual independent screening, formal meta-analysis) was not followed.

The search was primarily conducted in PubMed, Web of Science, and Scopus for articles published between January 2000 and December 2024. Key search terms included combinations as follows: ("inhalational anesthetics" OR "benzodiazepines" OR "ketamine" OR "propofol" OR "dexmedetomidine") AND ("PTSD" OR "post-traumatic stress disorder") AND ("fear memory" OR "fear conditioning" OR "fear extinction"). Studies were selected based on their relevance to the topic. Inclusion criteria were as follows: (1) original research articles (both human and animal studies) and high-quality reviews; (2) studies focusing on the effects of anesthetics on PTSD development, symptoms, or underlying fear memory mechanisms; (3) articles published in English. Exclusion criteria were as follows: conference abstracts, editorials, and studies with poor methodological quality.

During the analysis, we systematically extracted the following information: study authors and year of publication; study design (*e.g*., RCT, case report, animal behavioral experiment); key experimental interventions/manipulations; and major findings. The included literature was then synthesized, compared, and critically evaluated based on research themes and primary conclusions, with the aim of identifying consensus, controversies, research gaps, and future directions in the field.

## 3 INHALED ANESTHETICS

### 3.1 Inhaled Anesthetic

 Classic inhaled anesthetics, such as sevoflurane and isoflurane, are established to play a vital role in achieving sedation, analgesia, and muscle relaxation in clinical practice. These agents alter neuronal membrane fluidity, enhancing flexibility and disrupting ion channel function, thereby reducing neuronal excitability and inducing sedation and anesthesia [13]. However, their effects on acute (postoperation or ICU) and long-term (post-hospitalization) psychiatric outcomes—such as PTSD—in traumatic survivors remain unclear. This review summarizes current evidence regarding the impact of inhaled anesthesia on PTSD behaviors and underlying mechanisms. Key findings are summarized in Table **1**: Inhaled Anesthetic and PTSD.

#### 3.1.1 Animal Study

Sevoflurane acts through multiple molecular mechanisms, primarily potentiating γ-aminobutyric acid type A receptor (GABAAR)-mediated inhibition while suppressing excitatory pathways *via* N-Methyl-D-Aspartate (NMDA) receptor antagonism. A recent study illustrated that neonatal sevoflurane exposure in rats increases later stress susceptibility, impairing fear extinction due to enhanced GABAAR depolarizing activity in the developing brain [14]. Additionally, neonatal sevoflurane exposure reduced glutamatergic neuron activation in the basolateral amygdala during fear behavior, leading to diminished neural responses and impaired learning in adult mice [15]. Similarly, prolonged isoflurane exposure casues context-dependent fear memory deficits with downregulated NMDA Subunit 2B (NR2B) expression [16]. Collectively, these findings suggest that neonatal anesthetic exposure may cause long-term neurodevelopmental impairments, specifically in emotional learning (*e.g*., fear extinction and contextual memory). These deficits involve dysregulation of developing inhibitory (GABAAR) and excitatory (NMDA/glutamatergic) systems, which alter synaptic plasticity and neuronal activity in fear-processing regions like the amygdala.

 Additionally, studies implicate Akt/mTOR signaling and brain-derived neurotrophic factor (BDNF) in extinction learning [17-19]. Notably, sevoflurane preconditioning enhanced synaptic plasticity and reduced apoptosis *via* Akt/ mTOR activation in foot-shocked rats, suggesting therapeutic potential for PTSD [20]. Another study revealed that sevoflurane administration before (but not after) foot shock suppressed fear memory, potentially through BDNF modulation [21]. These findings reveal a paradox: sevoflurane disrupts developing fear circuits yet exhibits prophylactic efficacy in stress-exposed adults. This protective effect correlates with activation of extinction-associated pathways (Akt/ mTOR, BDNF) and enhanced synaptic plasticity—supporting its potential for alleviating PTSD-like phenotypes. Thus, the neurobiological consequences of sevoflurane exposure depend critically on developmental stage, exposure timing, and the activity of key neurotrophic signaling pathways.

Some studies suggest that isoflurane may confer neuroprotective effects in mice exposed to foot shock [22-24]. Notably, its suppression of fear conditioning varies with concentration, and the protective effect on fear memory expression is markedly dose-dependent. Isoflurane concentrations exceeding 0.67MAC are necessary to suppress diverse forms of fear conditioning [22, 24]. Conversely, frequent isoflurane exposure in infant mice increases stress sensitivity and fear extinction impairment in adult mice [25], revealing dual effects on PTSD susceptibility that depend on dosage, exposure duration, and developmental timing. Isoflurane thus exhibits a biphasic neurobehavioral profile: at therapeutic concentrations (>0.67 MAC), it suppresses fear conditioning and provides neuroprotection in stress-exposed adults, whereas repeated subanesthetic exposure during infancy induces persistent stress hypersensitivity and fear extinction deficits. This divergence highlights dose, developmental timing, and exposure duration as critical determinants of isoflurane's overall impact on fear circuitry.

#### 3.1.2 Clinical Trials

Sevoflurane and isoflurane are widely used in surgical procedures and ICU sedation. Postoperative PTSD is defined as PTSD that develops after surgery [26]. Notably, sevoflurane anesthesia has been associated with reduced incidence of postoperative PTSD in trauma patients [27]. Zhong *et al*. found that combined sevoflurane--propofol anesthesia resulted in better recovery and milder postoperative PTSD symptoms than propofol alone, suggesting sevoflurane’s neuroprotection potential. In ICUs, compared to intravenous sedation, isoflurane sedation may alleviate symptoms linked to PTSD [28], and isoflurane administration resulted in better neuropsychological outcomes after one year of ICU treatment [29]. Thus, inhaled anesthetics may be preferable to intravenous sedatives for minimizing adverse neuropsychological outcomes in ICU patients.

 Overall, inhaled anesthetics like sevoflurane and isoflurane may attenuate PTSD symptoms by modulating neurobiological pathways, including GABA/glutamate signaling, BDNF, and Akt/mTOR activity. Sevoflurane may reduce fear memory formation, but its effects are developmentally and temporally dependent, necessitating caution regarding long-term neurobehavioral consequences. Isoflurane’s efficacy is dose-dependent: higher concentrations suppress fear conditioning, yet prolonged or early-life exposure may be detrimental. Clinically, both drugs demonstrate advantages over intravenous sedatives in reducing PTSD-related symptoms, yet optimal dosing and long-term safety remain unclear. While these agents are promising for therapeutic use, further research is needed to address mechanistic inconsistencies and refine clinical applications.

## 4 INTRAVENOUS SEDATIVE

### 4.1 Benzodiazepines

 BZDs are a widely prescribed sedative-hypnotic drug valued for their anxiolytic, anticonvulsant, and sedative properties. They are frequently employed in clinical settings for preoperative sedation, anxiety relief, and as adjuncts in anesthesia to enhance patient comfort and relaxation. Their pharmacological action centers on the potentiation of gamma-aminobutyric acid (GABA), the principal inhibitory neurotransmitter of the CNS (central nervous system). By binding to the GABA_A_ receptor, BZDs enhance GABA’s ability to open chloride channels [32]. This increases chloride ion influx, leading to neuronal hyperpolarization and reduced excitability, resulting in sedation and anxiolysis. Here, we summarized the impact of BZDs on PTSD in preclinical and clinical studies. Table **2** Benzodiazepines and PTSD presents a systematic overview of key research findings.

#### 4.1.1 Animal Study

Animal studies reveal a complex and contradictory situation for benzodiazepines in PTSD-like models. Critically, timing and context appear pivotal: An animal study indicated that immediate administration of alprazolam after predator scent exposure interferes with the normal HPA-stress response and increases susceptibility to subsequent stress [33]. Additionally, administering low doses of alprazolam immediately after an electric foot shock in rodents has been associated with a worse response to other stressful situations, increasing the freezing response to trauma-cue exposure [34]. Together, these findings suggest a potentially negative effect when BDZs disrupt acute stress processing. Exposure to repeated inescapable electric foot shock during early life in rats induces anxiety-like behaviors, and nitrazepam does not significantly reduce most of these anxiety-related behaviors [35]. This lack of efficacy in a developmental stress model hints at limited utility for PTSD symptoms. However, these limitations are not consistently observed across studies. Low doses of diazepam reduced long-term behavioral deficits in mice that were exposed to electric foot shock, including freezing behavior when repeatedly exposed to contextual reminders and elevated anxiety levels [36]. This crucial distinction—potentially linked to post-consolidation timing or specific BDZ agents—suggests effects may be highly protocol-dependent. The contradiction between studies (*e.g*., alprazolam worsening outcomes *vs.* diazepam improving them) underscores that BDZ effects are not monolithic.

 Exposure therapy is based on fear extinction, which involves the suppression of conditioned fear responses. Critically, rodent studies reveal that BDZ timing critically determines extinction efficacy: In rodents, administering BZDs before extinction training can impair long-term extinction retention [37, 38]. This impairment likely stems from a paradox: while BDZs suppress amygdala hyperactivity (a therapeutic goal), they simultaneously inhibit amygdala-dependent inhibitory learning essential for extinction memory formation [38]. Equally concerning, BZD administration immediately after extinction training also impairs the consolidation of extinction memory [39, 40]. This suggests that BDZs exert detrimental effects regardless of administration timing. Recent mechanistic insights further complicate this narrative. Cheung *et al*. revealed the effects of an ultra-short-acting BZD, remimazolam, on the reuniens nucleus (RE) of the thalamus and the interconnected hippocampal-septal circuit during fear extinction. Systemic or RE-specific administration of remimazolam reduces RE activation through A-type GABA receptors, thereby impeding fear extinction [41]. This highlights that BDZs’ extinction-disrupting effects extend beyond the amygdala, involving distributed neural networks.

 Most preclinical studies investigating BZDs and fear extinction have been conducted in males. However, recent research has expanded to include female rodent models. Inhibition of the basolateral amygdala by infusing GABA_A_ agonists can impair fear extinction in nulliparous (reproductively inexperienced) female rats. BZDs may impair fear extinction in nulliparous females [42]. This effect appears consistent across reproductive states, as evidenced by a study examining diazepam administration timing in female rats. When administered either before or after extinction training, diazepam significantly impaired extinction retention in both nulliparous and primiparous females [43]. This uniform impairment across reproductive states challenges the assumption that hormonal status modulates BDZ effects on extinction, urging human studies to address sex-specific responses.

#### 4.1.2 Clinical Trial

 BZDs are widely used in clinical practice. A large number of systematic reviews and meta-analyses have evaluated the impact of BZDs on the occurrence and development of PTSD and their associated relationship.

PTSD patients often experience symptoms of anxiety and insomnia, so BZDs were initially used in the prevention or treatment of PTSD. A comprehensive review of pharmacological PTSD prevention found temazepam ineffective in reducing PTSD incidence following trauma exposure [44]. Similarly, Berger *et al*. concluded that, so far, there is no consistent evidence supporting the use of BZDs for the prevention or treatment of PTSD, although these drugs can alleviate some nonspecific symptoms such as insomnia or anxiety [45].

Alarmingly, emerging evidence inverts the risk-benefit calculus. Contrary to initial expectations, accumulating clinical evidence now identifies benzodiazepines as a potential risk factor for PTSD development. A previous systematic review suggested that BZD use and prolonged sedation in the ICU, alongside fear, stress, and delirium, might be risk factors for developing PTSD [46]. This concerning association has been further substantiated by two 2015 systematic reviews. The first indicated that BZDs should be relatively contraindicated for patients with PTSD or recent trauma, as they are ineffective for treatment and prevention and their risks often outweigh potential short-term benefits [47]. The second review and meta-analysis on PTSD in critical illness survivors found that BZD use is an ICU risk factor for PTSD symptoms [48]. This convergence of evidence from diverse cohorts suggests a class-effect hazard. Due to varying methodological quality, Campos *et al*. conducted a systematic review on this topic again [49]. Their meta-analysis of cohort studies revealed that BZD recipients had an increased risk of PTSD compared to non-recipients. Data from randomized controlled trials similarly suggested increased PTSD severity with BZD use. However, these conclusions were based on a few studies with poor to moderate quality, including meta-analyses that incorporated unreliable data and randomized controlled trials characterized by poor methodological rigor. Thus, while epidemiologic signals are concerning, Campos forces a sobering conclusion: causality remains unproven due to irreducible confounding.

 Exposure therapy is one of the most effective treatments for PTSD. Preclinical studies suggest BZDs may attenuate exposure therapy effects in anxiety disorders, with some clinical trials supporting this observation. In a randomized clinical trial involving 156 veterans with PTSD, it was found that alprazolam increased the incidence of PTSD when combined with virtual reality exposure [50]. One possible reason is that it reduces engagement and arousal, leading to a lack of improvement and subsequently increasing the likelihood of patient dropout [51, 52]. However, Melani *et al*. systematically reviewed the literature on whether BZDs disrupt the effectiveness of exposure-based interventions (EBIs) for treating PTSD and anxiety disorders. They found no conclusive evidence that BZDs impede the efficacy of EBIs [53]. Similarly, another RCT (randomized controlled trial) re-evaluated whether the use of BZDs is associated with a reduced response to exposure therapy and found that BZD use was not related to a weaker response to exposure therapy during either the treatment or follow-up period [54].

 Overall, it is evident that researchers hold various controversial views regarding the use of BDZs for the prevention and treatment of PTSD. At the very least, their safety and efficacy have not been recognized. The FDA's warnings emphasize the risks of misuse and dependence on BZDs. Currently, they are not considered suitable for the prevention or treatment of PTSD.

Some studies have explored this from a clinical mechanism perspective. Changes in BZD/GABAA receptor complexes play a role in stress and anxiety induced by fear memory. Animals exposed to harmful stimuli such as foot shock or forced swimming exhibited a 20%-30% decrease in BZD receptor binding in the prefrontal cortex and hippocampus [55]. The development of neuroimaging techniques such as positron emission tomography (PET) and single photon emission computer tomography (SPECT) allow for the determination of the distribution and density of single receptor molecules in the human brain [56]. In one study, SPECT imaging of BZD ligands revealed a reduction in BZD-GABA_A_ receptor availability in the prefrontal cortex of patients with combat-related PTSD. Another study used [^11^C] flumazenil and PET to assess differences in the BZD/GABA_A_ receptor complex between veterans with and without PTSD. Similarly, a general reduction in [^11^C] flumazenil binding was observed in PTSD patients [57]. This receptor downregulation implies that stress-induced GABAergic dysfunction creates intrinsic resistance to BZDs’ anxiolytic effects—a pathological adaptation that may explain their inefficacy and potential to exacerbate outcomes in neural circuits already compromised by trauma.

 The role of BZDs in PTSD remains controversial, with preclinical and clinical studies yielding conflicting results. While animal models consistently demonstrate BZDs' interference with fear extinction—particularly through amygdala-mediated pathways—important exceptions exist, such as diazepam inducing paradoxical reduction in long-term anxiety in shocked mice. These discrepancies may reflect dose-dependent effects, different timing of administration, or differences in stress paradigms. Clinical observations associating BZDs with increased PTSD risk remain confounded by indication bias, as more symptomatic patients typically receive these medications. RCT data present particular contradictions regarding exposure therapy outcomes: while some studies report compromised efficacy, others show no effect. This inconsistency may stem from heterogeneity in trauma types, treatment protocols, or assessment measures.

### 4.2 Ketamine

Ketamine, an N-methyl-D-aspartate (NMDA) glutamate receptor antagonist, inhibits excitatory neurotransmission, producing anesthetic and analgesic effects [58]. It also demonstrated efficacy in alleviating depressive and anxiety symptoms in depression disorders [59, 60] and PTSD symptoms [61, 62]. Beyond antidepressant actions, ketamine treatment in PTSD patients enhances synaptic plasticity, attenuates fear memory expression [63-65], and promotes fear extinction [66-68], thereby facilitating adaptive processing of traumatic memories and reducing PTSD symptom severity. Here, we summarized the impact of ketamine on PTSD in preclinical and emerging clinical evidence, with key findings summarized in Table **3**, Ketamine and PTSD.

#### 4.2.1 Animal Studies

Collective evidence reveals potential mechanisms underlying ketamine’s effects on PTSD. Here, we summarized three primary pathways: brain-derived neurotrophic factor (BDNF)-related pathway, mTOR-related pathway, and dopamine receptor modulation.

BDNF contributes to the pathophysiology of learning and memory disorders, including Alzheimer’s disease and PTSD [69-72]. Fear memory expression correlates closely with BDNF protein expression. For instance, the alteration of BDNF transcription can affect fear memory consolidation [73]. Moreover, the expression of BDNF is increased following extinction learning [74]. Ketamine activates postsynaptic glutamate signaling, elevating BDNF expression and promoting synaptic formation [75]. It also regulates fear memory through the GluN2B/BDNF pathway [76], highlighting this pathway’s vital role in ketamine administration in PTSD treatment. Furthermore, convergent evidence indicates that ketamine infusion protects synaptic integrity [75, 77, 78]. Glutamate receptor (GluN2B) dysfunction [79] or deletion [80] disrupts long-term fear memory, whereas NMDA receptor antagonists can increase the expression of BDNF *via* voltage-sensitive Ca^2+^ channels, activating cAMP phosphorylation response element-binding protein in humans. The BDNF-GluN2B interplay constitutes a core mechanism to fear memory modification: while BDNF dysregulation underlies impaired consolidation/extinction in PTSD, ketamine amplifies BDNF through glutamate surge and GluN2B activation. This dual action—restoring synaptic deficits while reprogramming fear memory—positions BDNF as both a pathological hub and therapeutic traget. Thus, targeting BDNF plasticity (*e.g*., *via* ketamine’s NMDAR antagonism) offers a promising strategy to correct fear circuitry dysfunction.

 The mTOR pathway regulates synaptic protein synthesis and mediates synaptic plasticity and fear memory formation [17]. Evidence suggests that the phosphorylation of mTOR's downstream target in the amygdala mediates fear response development [81]. Notably, ketamine activates the mTOR pathway increasing synaptic-associated protein expression and synaptogenesis [18, 82, 83].

 The dopaminergic system critically modulates fear memory and PTSD pathology [84]. Recent work indicates that ketamine ameliorates fear memory *via* dopamine projections from VTA to BLA and their receptors [63].

Overall, further study is needed to explore how ketamine affects stress-related behavior in the PTSD model.

Moreover, ketamine shows a two-sided effect depending on dosage in PTSD models. For instance, single 15 or 30 mg/kg ketamine administration after fear conditioning significantly inhibit fear memory retention, whereas 7.5 mg/kg does not [76]. An anesthetic dose enhances memory retention without altering memory strength in foot-shocked mice [85]. Notably, chronic ketamine infusion (20 mg/kg or 30 mg/kg for 28 days) downregulates NMDARs (*e.g*., GluA1, GluA2, GluN2A, and GluN2B), while 10 mg/kg spares GluN2B expression. Chronic exposure also reduces synaptic plasticity and transmission, leading to learning and memory impairment [86]. The dual effect of ketamine's behavioral impact depends critically on dose thresholds: single 15-30 mg/kg doses attenuate maladaptive fear memory, whereas chronic administration (≥20 mg/kg) severely suppresses NMDA receptor subunits (including GluN2B), impairing synaptic transmission and learning. Thus, ketamine functions as both a therapeutic agent and a potential iatrogenic hazard, necessitating precise dosing to achieve fear suppression while avoiding neuroplasticity impairment from prolonged supratherapeutic exposure.

#### 4.2.2 Clinical Trials

Clinical studies increasingly indicate the impact of ketamine on PTSD-related symptoms, positioning it as a promising intervention for PTSD prevention and therapy [62, 87]. Ketamine infusion shows significant preventive efficacy against PTSD development. Perioperative ketamine administration reduced postoperative PTSD incidence [88], and clinical evidence shows ketamine rapidly reverses structural and functional synaptic dysconnectivity in PTSD [77, 78, 89]. In an observational study of 603 burn patients, ketamine administration during treatment reduced PTSD prevalence [61]. Conversely, ketamine failed to prevent PTSD in acute military trauma [90] and even aexacerbated acute stress disorder symptoms in accident victims [91], underscoring its inconsistent efficacy in acute stress disorders.

Notably, accumulating evidence suppports ketamine’s efficacy and safety for chronic PTSD [62, 92, 93]. A randomized controlled trial reported that a two-week course reduced PTSD symptom severity in chronic patients [62], while a randomized crossover trial demonstrated that a single subanesthetic dose rapidly alleviated PTSD symptoms [92]. These findings substantiate ketamine's potential for chronic PTSD treatment, with greater therapeutic efficacy than observed in acute trauma injury [87].

The clinical paradox—ketamine’s effectiveness in chronic but not acute PTSD —requires re-evaluation of its therapeutic window. Prophylactic use benefits from pre-trauma neural circuit stability, while chronic treatment targets existing maladaptive synaptic patterns for remodeling. In contrast, acute trauma disrupts glutamate signaling that likely overcomes ketamine’s NMDA receptor blockade, shifting the excitation-inhibition balance toward harmful states. Therefore, ketamine’s efficacy critically depends on disease stage: it stabilizes consolidated pathological circuits but may disrupt ongoing pathological plasticity.

Dose and administration frequency critically influence ketamine’s clinical effects. Both single and repeated infusions alleviate PTSD-related symptoms and comorbidities (*e.g*., substance use disorder, depression, and reactive attachment disorder) in chronic PTSD patients [61]. An open-label clinical study demonstrated ketamine’s efficacy and safety for generalized anxiety disorder with PTSD comorbidity, effectively reducing symptoms with less major side effects [94]. Given its therapeutic profile for PTSD with comorbidities, ketamine represents a promising future treatment.

In summary, ketamine exhibits a complex therapeutic profile for PTSD, characterized by an inverted U-curve dose-response relationship requiring careful clinical management. Its efficacy derives from rapid synaptogenesis in the prefrontal cortex and reversal of stress-induced neuronal deficits, primarily mediated *via* mTOR and BDNF pathway activation. At optimal doses, these mechanisms promote fear extinction and restore synaptic connectivity, providing rapid relief for chronic PTSD symptoms. However, the therapeutic window is narrow—subtherapeutic doses imcompletely block NMDA receptors, inadequately modulating fear memory circuits and producing inconsistent fear retention effects. Conversely, excessive or prolonged high-dose exposure risks NMDA inhibition oversaturation, potentially impairing synaptic plasticity, inducing psychotomimetic effects, and worsening cognitive function, including memory deficits.

These divergent outcomes underscore ketamine's delicate balance between neuroplasticity enhancement and neural disruption. Clinical variability**—**driven by individual metabolic differences and PTSD pathophysiological heterogeneity**—**further complicates application, explaining mixed results across studies. Some trials report robust symptom reduction, while others note minimal benefits or even symptom exacerbation in acute trauma cases. This necessitates rigorously personalized dosing strategies that consider treatment timing, frequency, and cumulative exposure. Future research must optimize dosing parameters—balancing efficacy against long-term risks—while exploring adjunctive therapies to widen its safety margin. Currently, ketamine remains a promising but highly context-dependent intervention requiring cautious implementation to maximize therapeutic potential.

### 4.3 Propofol

 Propofol is a short-acting intravenous anesthetic widely employed for both inducing and maintaining general anesthesia. Due to its rapid onset and quick recovery, it is particularly favored in outpatient settings and procedures like endoscopy and minor surgeries. The mechanism of action of propofol primarily involves enhancement of GABA_A_ receptor activity, analogous to BZDs. Propofol binds to a distinct site on the GABA_A_ receptor, facilitating an increased influx of chloride ions when GABA is present. This leads to hyperpolarization of the neuron, resulting in decreased neuronal excitability and producing sedative, hypnotic, and anesthetic effects [96].

#### 4.3.1 Animal Study

 Propofol affects PTSD by modulating retention and consolidation processes of fear memory. Morena *et al*. observed that propofol induced persistent traumatic memory enhancement and anxiety effects in rats that were exposed to electric foot shock, potentially due to increased memory retention [85]. Another study indicated that anesthetic doses of propofol might regulate memory consolidation through the activation of the endocannabinoid system. In an inhibitory avoidance task, male Sprague-Dawley rats treated with propofol after training exhibited significantly increased latency in inhibitory avoidance performance at 48 hours, suggesting enhanced memory consolidation. Notably, this effect was abolished by rimonabant, a cannabinoid receptor antagonist, confirming the involvement of endocannabinoid signaling in propofol-induced memory modulation [97]. This implicates the endocannabinoid system as a novel mediator of propofol’s memory effects, distinct from GABAergic mechanisms of other anesthetics, yet this pathway remains unexplored in human PTSD.

 Additionally, emerging evidence suggests that propofol can influence fear memory consolidation and affect the development and progression of PTSD by impacting fear extinction memories. One study demonstrated that propofol has a certain reparative effect on impaired hippocampal long-term potentiation (LTP) in mice that are exposed to electric foot shock, while simultaneously facilitating fear memory extinction through the preservation of synaptic plasticity [98]. However, another study reported contradictory findings, indicating that propofol impaired the acquisition and recall of cue-induced fear extinction [99]. This discrepancy can be attributed to confounders like concurrent surgical stress (*e.g*., laparotomy) that synergistically disrupt extinction learning, highlighting the critical limitation of studying drug effects in isolation from clinical comorbidities.

 Unfortunately, current studies on propofol and PTSD have primarily focused on the phenotype, and the underlying mechanisms remain underexplored. Future research needs to delve deeper into the mechanisms to minimize the potential complications of PTSD associated with the use of propofol.

#### 4.3.2 Clinical Trial

 The majority of studies related to propofol and PTSD suggest that propofol has a promoting effect on the development and progression of PTSD. Usuki *et al*. investigated whether administering propofol within 72 hours of a motor vehicle accident (MVA) influences the development of PTSD symptoms. Using the Clinician-Administered PTSD Scale (CAPS) at six months post-MVA, they found that propofol administration might adversely affect PTSD development [100]. Similarly, Zhong *et al*.'s prospective study also indicated that the early use of propofol general anesthesia in emergency surgery may increase the risk of developing PTSD [27]. The literature on propofol use in the ICU reveals a more complex picture. A prospective study found that PTSD was associated with propofol use, dopamine administration, and hospital stay duration [101], while Zisopoulos *et al*. specifically linked PTSD symptoms to propofol administration duratio n [102]. However, a multicenter RCT by Strom *et al*. complicated this narrative by finding no difference in PTSD incidence between propofol/midazolam-sedated and unsedated mechanically ventilated patients [103]. This contradiction suggests that propofol's effects may depend critically on timing and clinical context. The coadministration of midazolam in the RCT may have also obscured propofol-specific effects through synergistic GABAergic actions.

 Recent studies have further investigated the potential mechanisms. Subclinical doses of propofol produce anterograde amnesia, manifesting as impaired early memory consolidation [104]. This raises the question: why does propofol fail to suppress the consolidation of fear memory? Pryor *et al*. used event-related functional MRI to study propofol's effects on the emotional memory system in humans. Their findings revealed that, at sedative concentrations, propofol was ineffective at inhibiting amygdala activation but eliminated emotional regulation and induced amnesia through reduced hippocampal reactivity. Thus, even if the memory of events is diminished, the amygdala-dependent fear system may remain intact [105]. Another study tested the effect of deep sedation on emotional memory reconsolidation in 50 participants. Propofol administered after memory reactivation disrupted reactivated memories, impairing subsequent emotional episodic memory retrieval [106]. This suggests potential therapeutic applications if precisely timed. However, pathological traumatic memories in PTSD patients likely involve fundamentally different consolidation processes compared to laboratory-induced memories in healthy subjects, highlighting the need for caution in translating these findings.

 In summary, current evidence regarding propofol's role in PTSD presents a complex picture marked by significant contradictions. Animal studies reveal propofol can both enhance traumatic memory retention through endocannabinoid pathways and facilitate fear extinction *via* hippocampal plasticity preservation—opposing effects likely attributable to variations in dosing, administration timing relative to trauma, and concurrent surgical stress. Clinical observations consistently associate propofol use with increased PTSD risk in trauma and ICU populations, though this may be confounded by illness severity and concurrent treatments, as suggested by the lone negative RCT finding. The mechanistic paradox—whereby propofol induces hippocampal-dependent amnesia while sparing amygdala-mediated fear responses—offers a plausible explanation for its failure to prevent PTSD despite impairing explicit trauma recall. Notably, propofol's capacity to disrupt emotional memory reconsolidation in healthy subjects presents a promising therapeutic avenue that remains unexplored in clinical PTSD. These contradictions underscore the need for more rigorous preclinical studies employing standardized PTSD models and clinical trials that control for baseline risk factors while investigating targeted applications, such as memory reconsolidation blockade. Until more evidence emerges, clinicians should remain cautious regarding propofol use in trauma-exposed patients while recognizing its potential dual nature as both a risk factor and future therapeutic candidate. Key findings are summarized in Table **4** Propofol and PTSD.

### 4.4 Dexmedetomidine

 Dex is a highly selective alpha-2 adrenergic receptor (α2AR) agonist widely employed in clinical anesthesia and sedation. Its pharmacological action involves the binding to α2AR in the central nervous system, thereby inhibiting norepinephrine release. This leads to a range of pharmacological effects, including sedation, analgesia, and anxiolysis [107]. Clinically, Dex is used for preoperative sedation, procedural sedation, and the management of patients in intensive care settings, where it provides effective sedation while maintaining patient arousability. Notably, its rapid onset and short duration of action enhance its utility in various clinical applications [9].

#### 4.4.1 Animal Study

 Conditional fear memory, as a typical model of traumatic memory, has been extensively studied, and its underlying mechanisms have been identified. These include the consolidation of fear conditioning, the instability/reconsolidation and extinction following the retrieval of fear memories, and the forgetting of fear memories [108]. Dex has been shown to modulate multiple phases of fear memory processing.

The role of Dex in preventing PTSD and alleviating its associated symptoms has been observed in clinical trials and pathological reports, corroborated by findings in animal models. Dex exerts preventive effects against PTSD by targeting early fear memory formation. As research continues to advance, the underlying mechanisms are also becoming clearer. Using a rat model of PTSD, which involved inducing the condition through five electric foot shocks followed by three weekly exposures to situational reminders, Ji *et al*. demonstrated that the stressed rats exhibited significant anxiety-like behaviors and cognitive impairments compared to the control group. Repeated administration of 20 μg/kg Dex led to reduced anxiety-like behaviors, impaired fear conditioning memory, and improved spatial cognitive function in stressed rats, compared to the vehicle-treated stressed rats [109]. This selective impairment of fear memory, while improving other cognitive functions, underscores Dex's potential to disrupt the core pathological memory processes in PTSD without causing generalized cognitive suppression. Another study evaluated the effects of ketamine, Dex, and propofol on fear memory consolidation and subsequent cognitive and emotional alterations related to traumatic stress exposure. The results indicated that Dex significantly impaired memory retention in the PTSD model without affecting long-term cognitive or emotional behaviors. Dex may have a preventive effect on PTSD [85]. Critically, and unlike some other interventions, this memory effect was achieved without inducing detrimental long-term cognitive or emotional alterations. This dissociation—impairing pathogenic fear memory while sparing general cognition and emotional state—is highly significant. It suggests Dex possesses a favorable therapeutic window for prevention, specifically targeting the traumatic memory trace.

 Fear extinction dysfunction is a notable feature of PTSD. In addition to its effects on fear conditioning and fear memory consolidation, Dex can also reduce fear by promoting the extinction of fear memories. Qing *et al*. examined the effects of propofol, sevoflurane, Dex, and surgery on fear extinction in adolescent mice. The results showed that Dex prevented surgery-induced impairment of cued extinction acquisition and recall but not consolidation [99]. This temporal specificity is crucial: it demonstrates Dex's ability to facilitate the active learning phase of extinction and the later retrieval of that safety memory. Further refining our understanding of Dex's contextual actions, Davies *et al*. [110] investigated its impact on discrete cue *versus* contextual fear conditioning. When administered before training, Dex selectively suppressed discrete cue fear conditioning. This suppression was accompanied by a decrease in memory retrieval-induced expression of c-Fos and P-CREB in the lateral, basolateral, and central nuclei of the amygdala. The accompanying neurobiological evidence provides compelling support for Dex impairing the encoding or very early consolidation of this specific fear memory trace. Crucially, this effect was absent in α2AR knockout mice, firmly establishing the essential role of α2-adrenoceptor activation. They concluded that Dex must be present during the encoding process to reduce discrete cue fear memory. However, its suppression of contextual memory is likely due to its ability to block the consolidation process. Their conclusion highlights a complicated, fear-type-specific mechanism of action that demands consideration in clinical application. Adding a vital layer of complexity, a recent study revealed significant sex differences in Dex's efficacy concerning fear extinction and anxiety reduction. Repeated administration of Dex accelerated the extinction of fear memory and reduced anxiety; it was found to be more effective in female rats compared to male rats [111]. This finding is of paramount clinical importance, as it suggests that the therapeutic potential of Dex for PTSD prevention or symptom mitigation may not be uniform across sexes. Ignoring this dimorphism could significantly impact the interpretation of clinical trial results and the design of future personalized treatment strategies.

Current research primarily focuses on behavioral phenotypes, with limited mechanistic understanding. The noradrenergic system plays a critical role in emotional regulation, particularly in anxiety, arousal, and stress. In PTSD patients, impaired α2AR-mediated inhibition coupled with enhanced noradrenergic activity may contribute to PTSD-like memories through increased noradrenergic signaling. Dex's potential preventive and therapeutic effects likely involve modulation of norepinephrine, a key neurotransmitter in fear memory formation [112, 113]. Similarly to Dex, clonidine is also an α2AR agonist. A study has shown that the α2AR agonist clonidine can reduce fear expression by weakening memory consolidation or reconsolidation, whereas the α2AR antagonist yohimbine can enhance the consolidation or reconsolidation of fear memory traces when administered immediately after acquisition or retrieval, resulting in increased freezing behavior [114]. As research deepens, researchers begin to focus on the role of specific brain regions. The bed nucleus of the stria terminalis is involved in the neural network regulation of emotions such as fear and anxiety [115], and it contains the highest density of norepinephrine in the brain [116]. Schweimer *et al*. revealed that injecting clonidine into the bed nucleus of the stria terminalis can significantly affect both learned and innate fear (anxiety) in rats. Clonidine injections interfered with both the acquisition and expression of fear-potentiated startle, as well as the enhancement of startle by light [117]. The locus coeruleus (LC) contributes to hyperarousal/hypervigilance, fear, agitation, anxiety, and panic attacks. These symptoms reflect heightened central and peripheral sympathetic activity by increased firing of LC neurons. Dex is predicted to reduce the firing of LC neurons in a dose-dependent manner, thereby mitigating the high sympathetic state [118]. Beyond these reported mechanisms, other molecular pathways remain unexplored.

#### 4.4.2 Clinical Trial

The translational promise of Dex for PTSD prevention has recently undergone significant clinical validation. Recently, a randomized controlled study published in JAMA Network Open indicated that administering Dex during and after surgery can reduce the incidence of PTSD in trauma patients. Dex might help decrease or counteract the early consolidation and development of conditioned fear memory [119]. This landmark finding provides the strongest clinical evidence to date supporting the preclinical hypothesis that Dex mitigates PTSD risk primarily by disrupting the early consolidation and pathological development of conditioned fear memory—a core mechanism identified in animal studies.

Beyond prevention, Dex demonstrates considerable clinical utility in managing a specific perioperative challenge intricately linked to PTSD: Emergence Delirium (ED) or Emergence Agitation (EA). ED/EA is defined as psychomotor agitation and delirium that typically occurs within 45 min from emergence of anesthesia [120, 121]. Over the past decade, there has been a sharp rise in PTSD among veterans. More veterans suffering from PTSD may require anesthesia and surgery to repair injuries and/or for treatment. The potential link between PTSD and ED has been suggested in the literature [122]. A previous study indicated that PTSD is a potential risk factor for ED in active-duty military personnel, highlighting the necessity for scientific research in this area [123]. The clinical efficacy of Dex for managing Emergence Delirium/Agitation (ED/EA) in patients with PTSD is supported by a compelling evidence base spanning different study designs and clinical approaches. Read *et al*. described three cases of ED in the post-anesthesia care unit in patients with a history of PTSD who were successfully treated with Dex [124]. Another case report indicates that military veterans with PTSD are more likely to have ED, and Dex infusion, along with verbal coaching, was effective in preventing ED for a veteran known to have “wild wake-ups” with prior anesthetics [125]. A randomized controlled study involving 369 patients with a history of military combat exposure who underwent general anesthesia and surgery indicated Dex proved to be effective in alleviating ED in combat veterans who exhibited preoperative anxiety symptoms. Anesthesia providers can safely use Dex to mitigate ED symptoms during the perioperative period [126]. This large-scale RCT elevates Dex as a strongly recommended strategy for perioperative ED mitigation, providing a crucial evidence-based foundation for clinical practice. Besides ED, EA is a phenomenon in the post-anesthetic period, and PTSD is closely associated with EA following anesthesia. Military veterans may be more susceptible to EA compared to the civilian population due to the high prevalence of PTSD among them [127]. Huang *et al*. presented two cases of successful EA prevention in patients with severe PTSD through the use of prophylactic Dex treatment preoperatively and intraoperatively [128]. Together, this evidence—from initial case reports to definitive RCTs and prophylactic applications—establishes Dex as a versatile, effective option for PTSD-related ED/EA throughout perioperative care.

Beyond its potential in preventing ED and EA, Dex demonstrates clinical benefits for PTSD patients undergoing complex surgeries [129]. Notably, Dex reduces requirements for supplemental sedation and analgesia while maintaining hemodynamic stability. PTSD is typically a relative contraindication for awake craniotomy because it may trigger the occurrence of PTSD. However, the intraoperative use of Dex allows patients to safely and smoothly pass the perioperative period without complications [130]. In psychiatric practice, pharmacologically assisted interviews serve as valuable diagnostic and therapeutic tools. A case report indicated that during a medication-assisted interview with two patients suffering from anxiety disorders, intravenous Dex was administered. The patients were able to actively participate in the interview without experiencing significant adverse events, and their recovery from anesthesia was quicker [131]. These findings position Dex as more than just a sedative, but rather a versatile agent enabling previously challenging interventions for PTSD patients. However, larger controlled studies are needed to optimize protocols and confirm efficacy.

 Overall, Dex has demonstrated significant potential for PTSD management through its unique modulation of fear memory processes. Preclinical studies consistently reveal its dual benefits: attenuating anxiety-like behaviors while disrupting fear memory consolidation through α2-adrenergic receptor-mediated mechanisms. Importantly, it enhances fear extinction—particularly in females—addressing a core deficit in PTSD pathophysiology. Clinically, Dex has proven effective in reducing PTSD incidence among trauma patients and managing Emergence Delirium/Agitation in military veterans, suggesting both preventive and symptomatic benefits. While its selective amnestic properties (affecting hippocampal-dependent memory more than amygdala-mediated responses) and perioperative study designs present some theoretical and methodological considerations, the accumulated evidence strongly supports Dex's role in regulating noradrenergic hyperactivity during critical memory consolidation periods. These findings position Dex as a promising therapeutic option, particularly for high-risk populations in the acute post-trauma phase. A summary of key findings is presented in Table **5** Dex and PTSD.

## CONCLUSION

PTSD is a debilitating mental health condition that develops following exposure to traumatic events involving actual or threatened death, serious injury, or violence [132]. In recent years, research has increasingly focused on the impact of anesthetic drugs on PTSD. This review examines the effects of commonly used sedative drugs on the development of PTSD. Dysfunction of the fear memory process is regarded as the core of various clinically prevalent psychological disorders among perioperative and critical care patients.

Current evidence from both clinical and preclinical studies reveals that anesthetics have complex and controversial effects on the fear memory system. Among these, ketamine and dexmedetomidine show promise in potentially mitigating fear memory. However, their positive efficacy can be influenced by factors such as dosage, timing, and individual susceptibility. Importantly, the evidence indicates that anesthetics may either ameliorate or aggravate PTSD symptoms, creating significant challenges for clinical application. The observed variability in outcomes, including numerous reports of null or paradoxical effects, emphasizes the necessity for more standardized research approaches before establishing definitive clinical guidelines. Given these nuanced and sometimes contradictory findings, this narrative review synthesizes evidence thematically to highlight key knowledge gaps and mechanistic insights (Fig. **1**), rather than adhering to systematic methodology. Further clarification of these variable effects—particularly through rigorous, quantitative meta-analyses—remains an important direction for future work.

Moreover, several key challenges persist in this research domain. First, fundamental studies have not sufficiently explored the underlying mechanisms, and identifying more specific molecular targets is crucial for improving drug efficacy and reducing side effects. Second, there is significant debate surrounding drug dosage and administration strategies, highlighting the need for further investigation to optimize these aspects and ensure clinical feasibility and effectiveness. Third, the long-term use of anesthetic sedatives could lead to tolerance, dependence, and other adverse effects. Finally, gender differences in the effects of anesthetics on PTSD warrant further investigation. Consequently, future therapeutic approaches should emphasize personalized treatment plans and consider combination strategies integrating anesthetic sedatives with other evidence-based interventions to develop more comprehensive and safer PTSD management options.

## Figures and Tables

**Fig. (1):**
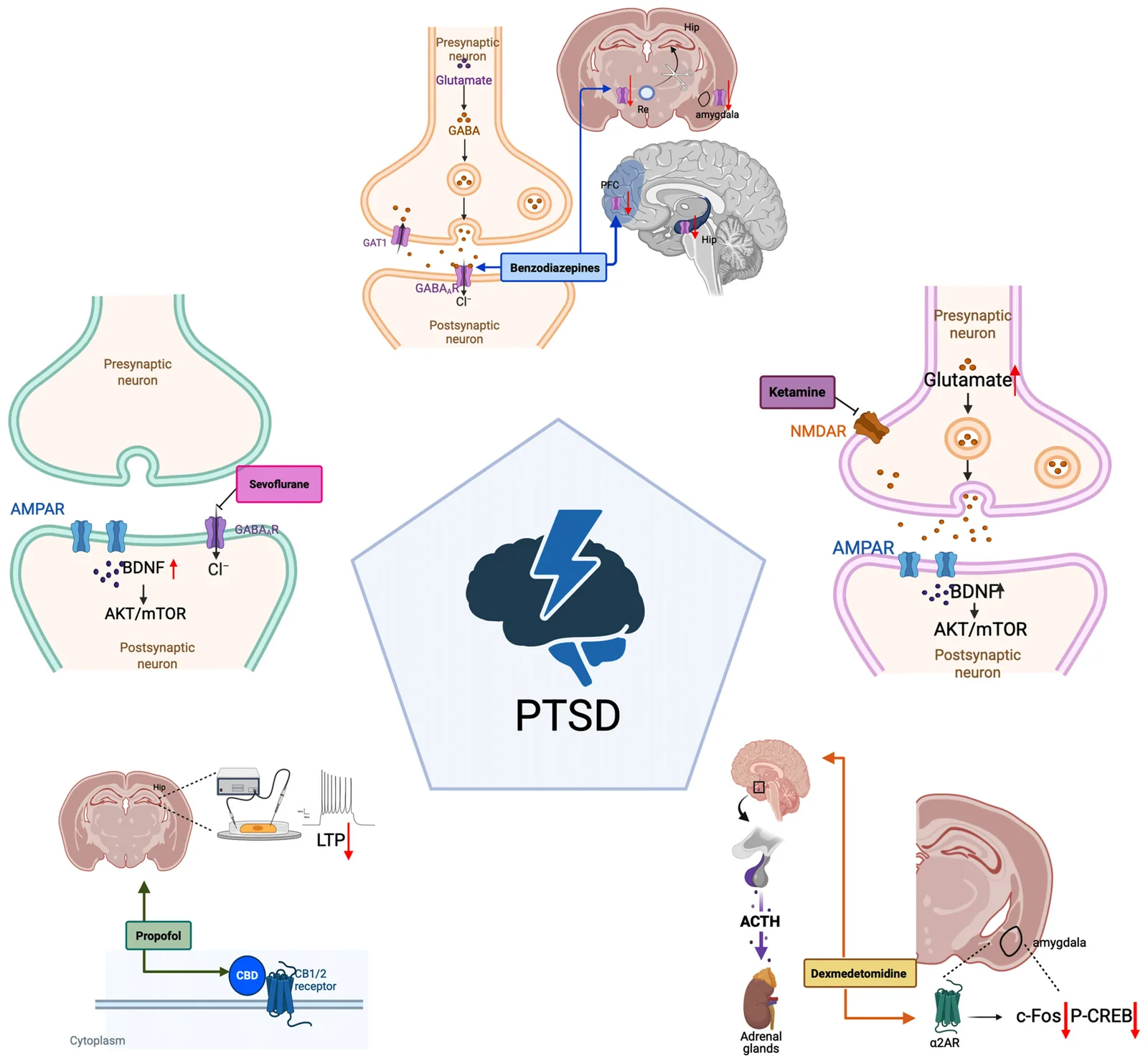
Illustration of the potential mechanisms by which five anesthetics influence PTSD. Sevoflurane enhances both GABA_A_R receptor depolarizing activity and promotes BDNF release with downstream mTOR signaling pathway regulation. Benzodiazepines act by potentiating GABA_A_ receptors, enhancing inhibitory neurotransmission in key brain regions like the amygdala, prefrontal cortex, and hippocampus. Chronic use may downregulate receptor availability and impair fear extinction by disrupting memory consolidation processes. Ketamine inhibits NMDA receptors and improves the release of glutamate. This glutamate then acts upon postsynaptic AMPA receptors, causing BDNF secretion. Subsequently, BDNF induces the mTOR mTOR-related pathway. Propofol modulates PTSD-related fear memory processes by potentiating GABA_A_ receptor inhibition and interacting with the CB1 receptor activation. Its effects on LTP in the hippocampus and amygdala further contribute to these divergent outcomes. Dexmedetomidine exerts its PTSD-modulating effects primarily through α2AR agonism, suppressing noradrenergic hyperactivity to impair fear memory consolidation/reconsolidation while enhancing extinction. Its actions in the amygdala, locus coeruleus, and bed nucleus of the stria terminalis normalize stress-induced hyperarousal and fear circuitry dysregulation.

**Table 1:** Inhaled anesthetics and PTSD.

-	Authors	PTSD Model Research Type	Drug Administration	Effect/Primary Endpoints
**Animal study**	[30]	Electric foot-shock	1.2% isoflurane or 2.4% sevoflurane for 6 hours	Isoflurane: no effect on fear memory consolidation; sevoflurane: impairing memory consolidation and retention
	[31]	Electric foot-shock	2.4% sevoflurane for 6 h	Impairing fear learning
	[20]	Electric foot-shock	2.4% sevoflurane + 98% O_2_ for 1 h	Improving PTSD-related behavior
	[21]	Electric foot-shock	0.8% sevoflurane administration for 30 min	Suppressing stress-enhanced fear learning
	[22]	Electric foot-shock	More than 0.5 MAC for 30 min	Suppressing fear conditioning to tone
	[24]	Electric foot-shock	0.4 MAC isoflurane for 1 h	Suppressing fear learning
	[25]	Electric foot-shock	1.4% isoflurane for 2 h, once a day for 3 days	Impairing fear extinction memory
**Clinical trial**	[27]	RCT	2-4% sevoflurane for anesthesia maintenance. during emergency surgery	Reducing the occurrence of postoperative PTSD
	[28]	RCT	/	Alleviating PTSD symptoms
	[29]	RCT	/	Better neuropsychological outcome

**Table 2:** Benzodiazepines and PTSD.

-	Author	PTSD Model Research Type	Drug Administration	Effect/Primary Endpoints
**Animal study**	[35]	Electric foot-shock	Clonazepam 0.5 mg/kg, PO	Did not relieve PTSD symptoms and reverse the underlying pathophysiology
	[36]	Electric foot-shock	Diazepam 0.25 mg/kg, IP	Reducing freezing behavior and anxiety levels, and decreasing behavioral deficits
	[38]	Electric foot-shock	Midazolam 1.25 mg/kg, IP	Impairing extinction but spares their re-extinction
	[37]	Electric foot-shock	Midazolam 1.25 mg/kg, IP	Impairing extinction learning
	[39]	Electric foot-shock	Midazolam 1.5 mg/kg and 3 mg/kg, IP	Disrupting the consolidation of extinction and contextual fear memory reconsolidation
	[40]	Electric foot-shock	Midazolam 3 mg/kg, IP	Disrupting fear memory reconsolidation and extinction consolidation
	[41]	Electric foot-shock	Remimazolam 2,4, 8 mg/kg, IP	Hindering conditioned fear extinction
	[42]	Electric foot-shock	Muscimol stereotactic brain injection (BLA), 2.63 nmol/3µL	Impairing fear extinction
	[43]	Electric foot-shock	Diazepam 1.0 ml/kg, SC	Impairing extinction retention
**Clinical trial**	[44]	systematic review	Temazepam 30 mg, PO at bedtime	No evidence to support the efficacy of temazepam in preventing PTSD onset
	[45]	Systematic review	Alprazolam, 1.5~6 mg/day, PO temazepam	No consistent empirical support for the prevention or the treatment of PTSD
	[46]	Systematic review	Lorazepam midazolam	Using of BZDs in the ICU are likely risk factors for subsequent PTSD
	[47]	Systematic review and meta-analysis	/	BZDs should be considered relatively contraindicated for patients with PTSD or recent trauma.
	[48]	Meta-analysis	/	Treatment with BZDs is associated with an increased likelihood of experiencing PTSD symptoms.
	[49]	Systematic review and meta-analysis	/	No definitive evidence that BZD use after traumatic events increases PTSD incidence or severity
	[50]	RCT	Alprazolam, 0.25 mg, PO	Diminishing the efficacy of exposure therapy, with more severe post-treatment symptoms and higher rates of PTSD
	[51]	Comparative study	/	Discontinuation of the BZD may improve exposure treatment results
	[53]	A systematic review of RCT	/	No definitive evidence that BZDs could hinder the EBIs' efficacy in treating PTSD and anxiety disorders
	[54]	RCT	/	Patients prescribed BZDs did not have a weaker response to prolonged exposure.

**Table 3:** Ketamine and PTSD.

-	Authors	PTSD Model Research Type	Drug Administration	Effect/Primary Endpoints
**Animal study**	[76]	Electric foot- shock	30 mg/kg, IP	Alleviating the fear of memory generalization
	[83]	Electroconvulsive Shock	10 mg/kg, IP	Increasing synapse and spine formation
	[63]	Acute social defeat stress	5 mg/kg, IP	Ameliorating post-traumatic social avoidance
	[85]	Electric foot- shock	125 mg/kg, IP	Long-term reduction of social behavior
	[86]	Electric foot- shock	30 mg/kg, 28 consecutive days, IP	Impairing learning and memory
	[95]	Electric foot- shock	10 mg/kg, IP	Facilitating fear extinction memory
	[18]	Electric foot- shock	10 mg/kg, IP	Alleviating fear extinction memory
**Clinical trial**	[78]	Cohort study	0.5 mg/kg intravenous infusion over 40 min	Restoring the connectivity between the PFC/subcortex and the rest of the brain
	[90]	Observational study	/	Ketamine administration was not a risk factor for the development of PTSD.
	[91]	Comparative study	Ketamine	Aggravating post-traumatic stress reactions during the acute trauma phase
	[88]	Observational study	Perioperative ketamine treatment	A lower prevalence of PTSD
	[92]	RCT	0.5 mg/kg intravenously	Alleviating PTSD symptoms for chronic patients
	[94]	RCT	(0.25, 0.5, 1 mg/kg administered subcutaneously) for one week	0.5-1 mg/kg ketamine administration can reduce anxiety disorder
	[62]	RCT	Six infusions of ketamine (0.5 mg/kg) over 2 consecutive weeks	Repeated ketamine infusions reducing symptom severity in chronic PTSD patients.

**Table 4:** Propofol and PTSD.

-	Author	PTSD Model/ Research Type	Drug Administration	Effect/Primary Endpoints
**Animal study**	[85]	Electric foot-shock	300 mg/kg, IP	Inducing persistent traumatic memory enhancement and anxiety effects in PTSD rats
	[97]	Electric foot-shock	250, 300, or 350 mg/kg, IP	Enhancing emotional memory consolidation
	[98]	Electric foot-shock	150 mg/kg, IP	Accelerating fear memory extinction
	[99]	Electric foot-shock	200 mg/kg, IP	Impairing the acquisition and recall of cued fear extinction
**Clinical trial**	[100]	RCT	Propofol is administered for ongoing sedation within the first 3 days	Using propofol in the acute phase might be associated with the development of PTSD symptoms 6 months later
	[27]	RCT	0.1~0.15 mg·kg^−1^·min^−1^ for anesthesia maintenance	Propofol administration in emergency surgery may increase the risk of developing PTSD
	[101]	Prospectively study	/	Recalled delusional memories and higher IES (Impact Event Scale) after discharge had a relationship with propofol
	[103]	RCT	Propofol is administered for ongoing sedation within the first 2 days	Propofol did not affect PTSD incidence
	[105]	RCT	Brain concentration of 0.90 μg /ml	had no amygdala-dependent fear system
	[106]	Comparative study	/	Propofol can mitigate aversive episodic memories by impairing reconsolidation

**Table 5:** Dex and PTSD.

-	Author	PTSD Model/ Research Type	Drug Administration	Effect/Primary Endpoints
**Animal study**	[109]	Electric foot-shock	20 μg/kg, IP	Impairing fear conditioning memory, improving spatial cognitive function and reducing anxiety-like behaviors
	[85]	Electric foot-shock	0.3-0.4mg/kg, IP	Impairing memory retention in the PTSD model without affecting long-term cognitive or emotional behaviors
	[99]	Electric foot-shock	20 or 40 µg/kg, IP	Preventing surgery-induced impairment of cued extinction acquisition
	[110]	Electric foot-shock	10- 20 µg/kg, IP	Suppression of contextual memory and block the consolidation process
	[111]	Electric foot-shock	20 and 40 μg/kg, IP	Accelerating the extinction of fear memory and reducing anxiety
**Clinical trial**	[119]	RCT	0.1 μg/kg/h, IV	Might help decrease or counteract the early consolidation and development of conditioned fear memory
	[124]	Case report	0.5-1 mcg/kg, IV	Have potential utility and efficacy to rescue therapy for adult patients with ED in the PACU
	[125]	Case report	75 mcg, IV in30 min	Dex infusion was effective in preventing ED
	[126]	RCT	1 μg/kg, followed by a 0.6 μg/kg/h, IV	Mitigating ED symptoms during the perioperative period
	[128]	Case report	20 mcg, IV	Preventing EA patients with severe PTSD

## LIST OF ABBREVIATIONS

ACTH: Adrenocorticotropic Hormone
AKT: Protein Kinase B (PKB)
AMPAR: α-Amino-3-hydroxy-5-methyl-4-isoxazolepropionic Acid Receptor
BDNF: Brain-Derived Neurotrophic Factor
CB1/2: Cannabinoid Receptor 1/2
CBD: Cannabidiol
c-Fos: Fos Proto-Oncogene
GABA: Gamma-Aminobutyric Acid
GABAAR: Gamma-Aminobutyric Acid Type A Receptor
GAT1: GABA Transporter 1
Hip: Hippocampus
LTP: Long-Term Potentiation
mTOR: Mechanistic Target of Rapamycin
NMDAR: N-Methyl-D-Aspartate Receptor
p-CREB: Phosphorylated cAMP Response Element-Binding Protein
PFC: Prefrontal Cortex
PTSD: Post-Traumatic Stress Disorder
RE: Reuniens Nucleus
α2AR: Adenosine A2A Receptor
